# Hospital and Institutional News

**Published:** 1920-05-15

**Authors:** 


### HOSPITAL AND INSTITUTIONAL NEWS.

### CENTENARY OF FLORENCE NIGHTINGALE.

One hundred years ago on Wednesday last was born Florence Nightingale, the great pioneer of nursing as it is to-day. Her great lessons were learned during warfare and it is due to the movement she inaugurated that nursing was brought to a stage of efficiency which went far to save many thousands of useful lives during the late war. Florence Nightingale's statue in Waterloo Place was a shrine which was the objective of many pilgrims, mostly nurses, as was the other and better statue in the crypt of St. Paul's. A large number of floral tributes to the memory of the great nurse were offered at both places, including, at Waterloo Place, a large chaplet from the Florence Nightingale Training School at St. Thomas's Hospital. Owing to the kindness of the Dean, the crypt of St. Paul's was thrown open without charge to nurses in uniform. At Westminster Abbey a memorial service was held on Centenary Day when the presence of a large congregation, including many matrons and nurses, testified to the fact that the memory of Florence Nightingale is yet green.

### THE STERILISATION OF MILK.

In their Special Report, Series No. 49, the Medical Research Committee has issued its findings on the renewed experimental work on milk sterilisation originally started in the Thompson Yates laboratories in 1914. The method is purely electrical and its technicalities need not be mentioned here, but it is worthy of note that milk can be rendered 99.93 per cent. free of *B. coli* and *B. tuberculosis* without raising its temperature higher than 63° to 64° C., that the keeping power is correspondingly increased and the taste unaltered. Work still remains to be done upon the true biological value of milk so treated, but it is stated that babies fed experimentally upon this milk have done extremely well. The committee concludes that their investigations have provided the country with "an elegant practical method of purifying milk for human consumption, of which the use upon a large scale now becomes a problem for closer financial and administrative examination." So good does the method seem to be that we hope those responsible for the present clean milk campaign are giving it their full consideration and also, as we see it expressed elsewhere, it will remain to ensure that adoption of the method leads to no relaxation of cleanliness in the byre and dairy.

### THE CLEANSING OF CUPS.

We are particularly glad to note that the Admiralty is making official arrangements that in future all glass, cups and tumblers, etc., used in the naval canteens are to be treated in boiling water immediately after employment for drinking purposes. Earlier similar provisions for sterilising had been made for conditions of service afloat, and simple though this measure appears to be, we have no doubt it will materially reduce the incidence of sputum-borne diseases in the naval service. Some-time ago we drew attention to the dangers of drinking-vessel infection during ordinary normal civilian life. Among many small hygienic reforms we have already noted improvement in most of our reconstructed refreshment venues, and if any should doubt the urgency of this matter we refer them to "The Poison in the Cup" as a correspondent aptly termed his article (May 31, 1919, p. 208).

### MOTOR-CAR TAXATION.

We note with satisfaction that, during the reception of the B.M.A. deputation at the Ministry of Transport, Sir Henry Maybury, Chairman of the Taxation Committee, stated that it was not proposed to place any taxation at all on motor-ambulances. Otherwise, the possibility of allowances for the medical profession itself does not look hopeful. It was pointed out that if any exceptions were made to the general abolition of rebates, other classes also would claim special treatment; also that every commercial vehicle was now laid under tribute. The statement that the new taxation would enable road improvements to be made to the general benefit of motor users is obviously true in the main, but appears to us to have singularly little bearing upon the position of a doctor who must of necessity keep two medium-powered cars in commission. The excessive wear and tear on the modern roads is undoubtedly due to the disproportionate increase of the heavy commercial vehicles which use them, and the liability of their owners should be very considerably greater than that of a doctor, whose medium-powered car suffers on these roads, proportionately, far more damage than it causes, and this in the service of the local community.

### SIR ARTHUR STANLEY'S CONFIDENCE.

There was a distinct note of hope that the voluntary system would triumph over all difficulties sounded at the annual dinner of the Incorporated Association of Hospital Officers, held at Frascati's restaurant on Friday, May 7. Before any of the toasts of the evening had been proposed the gathering, following the lead of the President, Mr. J. H. Shaw, Secretary of Southport Infirmary, rose silently in their places as a tribute to the memory of Sir Henry Burdett. Both the President and Sir Arthur Stanley, who was the guest of the evening, stated in simple phrases that the inspiration of Sir Henry's life, and particularly his recent work, should be a great source of strength to all hospital workers during the present anxious and difficult times. Sir Arthur humorously described himself as the lamb in the midst of lions—the hospital treasurer surrounded by hospital secretaries. He said that many hospital treasurers at the present time either had the "wind up" or were victims of neurasthenia. The hospitals were suffering from an accumulation of ailments, from the rise in prices, from the increase in rates of wages, from the accumulation of necessary repairs delayed by the war. He could not but think that the crisis was temporary and had absolute belief in the public of the country and their generosity. The bank balance of the Red

Cross was £9,000 when the war started, and since then the public had given £19,000,000 for its operations. In America the Red Cross funds of £187,000 had increased to £19,000,000. It was but necessary to show that a cause was a good one and to succeed in reaching the individual giver and all would be well.

### POSSIBLE STATE ACTION.

On the night before the Hospital Officers' dinner Dr. Addison, answering a question in the House of Commons, said that he had been approached by certain hospital authorities and he was considering what was best to be done to help the hospitals through their present troubles, but a very wide issue was involved. Sir Arthur Stanley referred to such possible State aid, but said that it would be a calamity if the voluntary hospitals came permanently within a system of State subsidy. He said that there are three alternatives. The first is entire State support and management; this he knew Dr. Addison is, and the late Sir R. Morant was, entirely opposed to. The second is rate-supported institutions; these are not likely to be satisfactory and are certain to be unpopular, as the rates are quite high enough as it is. The third alternative is a continuance of the voluntary system, and to that he intends to devote his entire energies. Only ten per cent. of the population contribute to hospitals at present; with careful and thorough work another twenty per cent. should have the benefits of the privilege of giving to the sick brought home to them. The Red Cross intends to make a very great effort on these lines, and he hinted that although a start had been made first in the provinces, London is not to be left out. Sir Arthur Stanley said that he thought most strongly that the present competition for funds of hospital against hospital must cease, and the appeal must be collective. On these lines the £210,000 a year needed for London would be forthcoming.

### A JOURNALISTIC MYSTERY.

Some reference was made at the dinner to the letter to *The Times* of Mr. S. G. Asher. His main suggestion that a committee of chairmen of general hospitals should be formed to watch the situation was at least new, and and as a contribution to the debate in *The Times* is worthy of consideration. Why special hospitals should have no voice is not explained. In any case Mr. Asher's suggestion, or something like it, has already been carried out, and as was announced at the dinner, the British Hospitals' Association has formed local committees, including one for London, chosen from the very representative list of members of the Association. These committees will hold a watching brief on behalf of the hospitals (as would Mr. Asher's committee of Chairmen) and will be able to take action pending the calling together of the larger body of members. With so many important developments of the situation likely to show themselves at any moment the action of the British Hospitals' Association is one which will commend itself to hospital workers, who are all following the course of events with earnest attention.

### SIR H. BURDETT AND NURSE REGISTRATION.

The attitude of Sir Henry Burdett upon the question of the registration of nurses was always consistent. This consistency was well shown in the article published last week in The Hospital by Mr. G. Q. Roberts, C.B.E., Secretary of St. Thomas's Hospital, who, before his appointment to that post, had once been a member of Sir Henry's personal staff. His interesting recollections contained the following passage, which we reprint from The Hospital of May 8, page 145, column 2:—

> He [Sir Henry Burdett] never shrank from entering upon a controversial dispute. He took an active part in the bitter controversy in regard to the registration of nurses. In the earliest days of this movement he published a Register of Nurses, but the leaders of the nursing world, women who were devoting their lives to the betterment of the training and the improvement of the status of the trained nurse, convinced him that the time for registration had not yet come. In his weekly paper he gave the strongest encouragement to forwarding all improvements in the education and training of nurses, believing that registration should come in due time when a standard of registration acceptable to the majority had been established. The foundation of the College of Nursing, which has enlisted the sympathies of practically all the leading matrons of the hospitals and infirmaries of Great Britain, received his warmest support in this paper and in his journal, *The Nursing Mirror*, which has for always carried great weight with nurses.]

This evidence should not be buried. It is due alike to his subject and to Mr. Roberts himself to represent the above passage as an independent record of a policy which, because it concerned a controversial question, has not been adequately understood.

### THE LATE SIR RATAN TATA.

The recent death of the Parsi philanthropist, Sir Ratan Tata, did not evoke much notice in the newspapers, except as regards his rather remarkable will, under which from estate valued at £1,381,507 he left large legacies to many institutions in India. He made ample provision for his wife and family, and expressed the earnest desire that his wife should not wear mourning for more than a year after his death, and then should move about and mix in society as she had been doing in his lifetime, and should "dress and deck herself quite regardless of the custom to the contrary prevailing among the Parsis." A reversionary bequest was made for the advancement of education, learning, and industry in all its branches, including economy, sanitary science, and art, or for the relief of human suffering, or in works of public utility. The name of Ratan Tata is well known to those training as hospital almoners, for there is a Ratan Tata Department of Social Science and Administration connected with the London School of Economics and Political Science which, owing to the endowment of the late Sir Ratan Tata, offers facilities, probably unique of their kind, for the practical as well as the theoretical training of professional and voluntary social workers.

### AMERICAN HOSPITAL IN LONDON.

Considerable progress has been made in the plans for the American Hospital in London, which is expected to be opened before the end of the year. The list of officers is brilliant, Mr. Taft, as foreshadowed in our issue of April 19, has accepted the office of President in America; Lord Reading is President in England, with Lord Bryce as Vice-President. A committee has been formed of American residents in London, which includes many well-known names. It is hoped that in the autumn a temporary building for patients will be opened. As has been already indicated, it is intended that the institution shall be the principal research centre in Europe for American graduate students. The Committee in this country includes Sir Arbuthnot Lane, Sir Humphry Rolleston, Sir John Bland Sutton, Sir John MacAlister, and Mr. Philip Franklin. The Committee in the United States, which will work in conjunction with the English body, consists of Dr. George W. Crile (nominated by the American Academy of Science on International Relations), Dr. W. J. Mayo and Dr. Charles H. Mayo of Rochester, Dr. A. H. Ochsner of Chicago, Dr. Rudolph Matas of New Orleans, and Dr. Franklin Martin of Chicago. Mr. Philip Franklin, the Honorary Organising Secretary, states that a campaign will be opened to raise an endowment fund of several million dollars.

### FOOTBALL CLUBS AND HOSPITALS.

In our issue of May 1 we referred to the fact that the majority of the big professional football clubs do not subscribe a great deal to hospitals. We have since had brought to our notice an exception in this respect. At Norwich in 1904, a number of working men subscribed about £50 for the purchase of a silver cup, to be played for annually by Norwich City and a team to be invited by a committee of football enthusiasts appointed to govern the competition. Several of the English League clubs have visited Norwich to play for this cup since 1904, and the Norfolk and Norwich Hospital had benefited to the end of 1919 by the sum of £1,771 from the gate money taken at the annual matches. This season a big effort was made to make up for the war-time period, when no matches could be played, and the committee were fortunate in inducing Tottenham Hotspur to visit Norwich on Saturday, May 8, to play Norwich City. A crowd of twelve thousand attended the match, and it is anticipated that the hospital will benefit, after the travelling expenses of the visiting team and the provision of gold medals for the players have been paid for, by over £700. The secretary of the Norfolk and Norwich Hospital, who represents the hospital on the committee, organised, with the assistance of three of the members, the sale of flags amongst the public attending the match, and this realised £123.

### THE BRITISH HOSPITAL AT MARSEILLES.

The first stone of the hospital for sick and wounded British seamen at Marseilles was laid on May 2 by Lady Burghclere, the architect of the new building being Mr. Barnard. The president of the committee is Mr. Vicars, the British Consul-General, and the project is warmly supported by the English colony and the agents of British shipowners at Marseilles, and, thanks to the devotion of Lady Burghclere, whose husband is a director of the Peninsular and Oriental Company, a strong financial committee has also been formed in London. In addition to the British colony, the city of Marseilles has evinced practical sympathy and encouragement. The hospital will contain all modern requisites, will be provided with sixty-four beds, and is intended to afford treatment for all diseases. The site has been admirably chosen, being quite near the port, but at the same time in a rural road. The committee has already finished the preliminary work in the way of giving access to the building and the arrangement of the ground and drains, and the actual erection should now proceed without delay. Queen Alexandra sent a message, which was read at the ceremony of laying the foundation-stone, saying how much we owe to the brave men of the Mercantile Marine, especially for their devoted service during the war.

### SECRETARY'S WIDOW RECEIVES PENSION.

We understand that the Committee of the Cancer Hospital, Brompton Road, has granted a pension of £300 a year to the widow of their late Secretary, Mr. Fred W. Howell. The news of this generous provision will be received with much satisfaction by workers in voluntary hospitals generally and by Mr. Howell's many friends in particular. What to do in respect of the family of an officer who dies before retirement is always a difficult problem, and in several instances brought to our notice the solution has been the granting of one year's salary to the widow, a gift which, while it enables her to "look round" or to augment other sources of income, cannot be considered as a permanent means of support. Secretaries and other hospital officials have seldom, from their modest emoluments, been able to make adequate provision for old age or for their families should they die in working years. Reliance upon the general practice, in the absence of a regular pension fund, of granting pensions, based on the merits of each case, has been the hope of most men. The fact that a pension has been saved may weigh with many governing bodies, but how far the theory can be applied by trustees is an uncertain quantity. Nevertheless, few will find fault with the generous decision of the Committee of the Cancer Hospital.

### BUTTER SUPPLY OF RATIONED OFFICERS.

Lambeth Board of Guardians have had to deal with a serious problem, due chiefly to the fact that their officers are still on short rations, and that no steps have been taken to bring the dietary scale to anything within pre-war limits now that the shortage of foodstuffs is not so acute. The District Auditor has discovered that certain articles of controlled food, particularly butter, which was intended for inmates and patients, have been consumed by the officers. Mr. C. W. Baldwin said that 1,000 lb.

of butter had been obtained in twenty-six weeks for the inmates, and used by the officers, whilst Mr. Frank Briant, M.P., acknowledged that the patients did have butter, but they did not get it all. Throughout the rationing period the guardians have been most careful that everybody in their institutions should be treated alike, and this extraordinary discovery came to them as a great surprise. The responsible officers have been reprimanded, and it is to be hoped that in future there will be no recurrence of such a regrettable incident.

### AMALGAMATIONS IN THE AIR.

The scheme for amalgamating Bristol hospitals, as will be remembered, includes the Royal Infirmary, the General Hospital, the Children's Hospital, the Orthopædic Hospital, the Cripples' Home, and the Eye Dispensary. On May 31 representatives of these institutions will be invited to pass such resolutions as may be necessary to the attainment of this end. The Earl of Shaftesbury, speaking at the annual meeting of the Belfast Ophthalmic Hospital, alluded to its proposed amalgamation with the Benn Ulster Hospital and, subject to the overcoming of any legal difficulties, strongly supported the measure. Local general opinion seems to be that amalgamation would be a great advantage. Lord Shaftesbury said that as hospitals had to pay legacy duty it would be a great saving if in their lifetime their friends could make up their minds to give to charities what they intended eventually to leave to them.

### THE SUPERVISION OF DEFECTIVES.

In order to exercise supervision over certain mental defectives, it is desirable that reports should be submitted from time to time as to the conditions and circumstances of their homes. The Health Committee of the Hertfordshire County Council considers that this information could be obtained by the various nurses in the county who, in their ordinary duties, frequently come into contact with defectives. As the Committee points out, these reports would contain information with reference to the home conditions of the defectives, or to the manner in which they are fed and clothed and protected from danger, and as to the extent to which they are efficiently cared for and supervised by their parents or guardians. In those districts where county council health visitors are employed, this information would be supplied without cost, but in those districts where the health visiting and nursing are undertaken by the nurses of the District Nursing Associations, a fee will have to be paid for obtaining these reports. Hence the Committee suggests a fee of 2s. 6d. for each initial report and 1s. for each subsequent visit.

### WORSE OFF THAN UNSKILLED LABOURERS.

The annual report of the Hull Asylums Committee states that the conduct of nurses and attendants has been on the whole satisfactory and in most cases highly commendable. The improvement in wages and other conditions of service does not, however, seem to attract a better class of candidate. The wages offered compare unfavourably with those now paid to unskilled labourers in other departments, and it is almost impossible at present to get candidates of any kind.

### UNMARRIED WOMEN IN MATERNITY HOMES.

Dr. Townend, the medical officer of the Hull Maternity Home, has asked the Corporation Health Committee whether it would be willing to admit unmarried women when special circumstances seem to justify help being given. She points out that all the larger homes in other cities admit such cases for a first confinement, but refuse help for a second. Those who have experience of the working of such homes find no difficulty in associating the two classes of women. To admit unmarried women would be to add very considerably to the value of the home as a training school for the nurses because it would increase the proportion of first confinements, which are far more valuable for teaching purposes than the others. The Committee has agreed to admit unmarried women in special circumstances, but for first cases only, and provided that a ward can be set aside for this class of case. A report is to be made six months hence giving the result of the experience gained in relation to the admission of unmarried women, so that the decision may, if necessary, be reviewed.

### SARAH GAMP IN THE MINORITY.

Durham County Council is able to report that for the first time since the inception of the Midwives Act, the number of trained practising midwives exceeds the number of untrained women in practice. The county midwives inspector mentions that the midwives, especially those who have recently trained, often express a wish for the opportunity of attending lectures to enable them to keep up to date and to discuss their work with other midwives. Such facility for enlarging their knowledge ought to be provided, and it is hoped that appropriate lectures by medical practitioners may be arranged for suitable centres in the near future.

### THIS WEEK'S DRUG MARKET.

There is still no improvement in business, and buyers seem more disposed than ever to postpone their purchases as much as possible in the hope that the downward movement of prices will become more comprehensive. The yield of cod-liver oil from the Norwegian fisheries this season has been extremely good, and supplies are available at substantially lower rates than those experienced in recent years. Among the articles that have moved downward in price are cardamoms, ipecacuanha, phenazone, star aniseed oil, barbitone, clove oil, guaiacol carbonate, resorcin, cascara sagrada, almond oil, lemon oil, and balsam tolu. Generally speaking, the decline is not substantial, but the downward movement is distinctly noticeable. The price of Japanese refined camphor has moved upwards again, but it is still substantially below the high figure that was ruling until recently. At the recent public sale of drugs held in Mincing Lane the demand was extraordinarily quiet and an unusually large proportion of the offerings was bought in the absence of bids.

